# The Emerging Role of the Gut Microbiome in Cerebral Cavernous Malformation: A New Novel Therapeutic Strategy?

**DOI:** 10.3390/ijms26178622

**Published:** 2025-09-04

**Authors:** Hamidreza Sadegh, Jaesung P. Choi

**Affiliations:** 1 Centre for Inflammation, Faculty of Science, School of Life Sciences, Centenary Institute and University of Technology Sydney, Sydney, NSW 2007, Australia; 2 School of Biomedical Sciences, UNSW Sydney, Kensington, NSW 2052, Australia

**Keywords:** gut microbiome, gut–brain axis, cerebral cavernous malformation, CCM, diet, probiotic, prebiotic, synbiotic

## Abstract

Cerebral cavernous malformation (CCM) is a cluster of abnormal blood vessels in the brain that leads to severe neurological deficits, seizures, and fatal hemorrhagic stroke. Currently, there is no available drug treatment for CCM. Most CCMs are conservatively managed by observing change in appearance (MRI), recent hemorrhage, or any clinical symptoms. Neurosurgery is the only current treatment option, but it is only effective in a few cases. Since most CCM lesions are surgically inaccessible, when left untreated they lead to severe neurological deficits, seizures, and fatal hemorrhagic stroke. Hence, new non-invasive, safe, and effective treatment strategies are urgently needed. Recent research has identified gut microbiome dysbiosis and its innate immune response as the critical stimulus in experimental CCM pathogenesis, demonstrating the importance of the gut–brain axis in CCM. Importantly, CCM patients also manifest gut microbiome dysbiosis and gut barrier health can impact CCM disease course. This review highlights the emerging involvement of the gut microbiome in CCM pathogenesis and its potential as a therapeutic target. While preclinical data suggest mechanistic links, the lack of clinical intervention studies limits current applicability and underscores the need for translational research.

## 1. Introduction

Cerebral cavernous malformation (CCM), also known as cavernoma and cavernous angioma, is a cluster of vascular anomalies of the central nervous system (CNS) [1], affecting approximately 35 million individuals globally each year, with a prevalence of around 0.5% [2,3,4]. Importantly, CCM is one of the commonest vascular malformations causing hemorrhagic stroke in children and young adults [5]. These vascular anomalies develop into mulberry-shaped cavities and form lesions in the brain. These vessels are predisposed to undergo acute intracranial hemorrhage and subclinical bleeds, and they can lead to severe headaches, seizures, hemorrhagic stroke, and neurological deficits [5]. The exact prevalence of CCM is unknown because up to 40% of affected individuals are asymptomatic [5]. However, autopsy studies estimate the prevalence of CCM to be between 0.2% and 0.5% of the population [6].

Ultrastructural analysis shows that CCMs are composed of abnormal cystic vascular channels lined by a single layer of disorganized endothelial cells (ECs) having impaired tight junctions [7,8,9]. The only treatment is neurosurgery; but this is only possible when lesions are surgically accessible and carries substantial risk of permanent neurosurgical side effects. Consequently, there is an urgent need for non-invasive treatments, which require the identification of druggable targets and development of therapeutic interventions.

CCM is inherited as a dominant disorder due to loss-of-function mutations in one of three genes: CCM1 (KRIT1), CCM2 (Malcaverin, Osm), or CCM3 (PDCD10) [10,11]. CCMs appear in sporadic and familial forms. Sporadic CCMs typically present as single, isolated vascular lesions, whereas familial CCM is characterized by the gradual development of multiple lesions over time due to inherited mutations in CCM genes. Despite these differences in clinical presentation, both forms of the disease converge on a common pathogenic mechanism: dysregulation of the highly conserved MEKK3-KLF2/4 signaling pathway. Under normal physiological conditions, this pathway plays a central role in maintaining endothelial quiescence and vascular integrity by responding to laminar shear stress and regulating anti-inflammatory and barrier-stabilizing gene expression. In the context of CCM, loss of CCM gene function leads to aberrant activation of the MEKK3–KLF2/4 axis, resulting in excessive endothelial proliferation, loss of vessel stability, and increased permeability, ultimately driving lesion formation and progression [12,13]. Patient prognosis is typically unclear, as lesions can rupture unexpectedly, suggesting that non-genetic factors (e.g., microbiome, inflammation) play a critical role in its progression [14,15].

The clinical manifestations of CCM disease are highly variable, ranging from mild symptoms such as headaches and weakness or numbness to more severe outcomes like seizures, focal neurological deficits, and stroke [16,17]. This variability can occur even among individuals with the same genetic mutations and family history [18]. Some individuals may experience a stroke in childhood, while others remain asymptomatic throughout their lives. In addition to genetic mutations, recent studies have identified and demonstrated that the role of modifiable factors such as the gut microbiome can modify CCM disease course [14,15,19,20].

The development of CCM lesions requires the accumulation of multiple genetic and non-genetic factors, which is hypothesized by a “third-hit” mechanism [21]. The first hit is an inherited germline mutation [10,11] and the second hit is a somatic mutation acquired during an individual’s life time [22]. The third hit is a non-mutational third parameter that is needed in addition to germline and somatic mutations to cause a phenotype [23]. Non-genetic factors such as inflammation, radiation exposure, hypoxia and oxidative stress can all interact with the genetic mutations that predispose individuals to CCM, ultimately facilitating the development of brain lesions [23]. However, it is important to consider that while non-genetic factors can contribute to CCM lesion formation and disease severity, they are not always identifiable in all individuals having CCM.

## 2. Gut Microbiome in CCM Pathogenesis

Changes in the gut microbiome are associated with many human diseases but in many cases, specific mechanisms are not yet well defined [24]. Similarly, the gut microbiome has emerged as a player in the progression of various cerebrovascular diseases [25,26,27] including CCM disease [14,19,20].

The role of the gut microbiome in CCM was first identified by Tang et al. in 2017 [19]. This study in mouse models showed the gut microbiome via endothelial toll-like receptor 4 (TLR4) and its innate immune response as critical inducers of CCM, demonstrating the existence of an aberrant gut–brain axis in CCM pathogenesis [19]. Specifically, the study showed that activation of TLR4 by Gram-negative bacteria (GNB) or lipopolysaccharide (LPS) accelerated CCM formation in mice by activating the MEKK3-KLF signaling pathway [19]. This was further tested and demonstrated, as genetic and pharmacological inhibition of TLR4 signaling prevented CCM formation in mice [19]. Antibiotic use can significantly alter gut microbiota composition, potentially favoring the overgrowth of LPS-producing Gram-negative bacteria. For example, studies have shown that treatment with antibiotics such as vancomycin or ciprofloxacin may reduce commensal populations and promote LPS-producing *Enterobacteriaceae* expansion, thereby increasing the systemic endotoxin level [28,29]. However, the load of the GNB *Muribaculaceae* (aka *Bacteroidetes* s24-7) is increased in mice having CCM lesions, and a single course of broad-spectrum antibiotic treatment or germ-free environment prevented CCM formation [19]. Furthermore, the role of TLR4 pathways is also suggested in humans, as polymorphisms that increase the transcription of the *TLR4* gene or its co-receptor CD14 were associated with higher CCM disease severity and burden in humans [19].

Further supporting the evidence that the gut microbiome plays a key role in CCM pathogenesis, a subsequent animal study demonstrated that disruption of the gut barrier is a primary determinant of CCM disease severity [20]. Chemical gut barrier disruption with dextran sulfate sodium (DSS) augmented CCM formation in mice [20]. Further, loss of Mucin-2 or exposure to dietary emulsifiers to reduce the mucus barrier increased CCM burden [20]. Conversely, treatment with the anti-inflammatory corticosteroid dexamethasone inhibited CCM formation in mice due to the combined effects on both brain ECs and gut epithelial cells [20]. Although specific studies examining gastrointestinal physiology in CCM patients are lacking, increased intestinal permeability resulting from gut dysbiosis could elevate circulating LPS, activating endothelial TLR4 signaling and promoting CCM lesion development, as demonstrated in murine models. Investigating gut barrier function and motility in CCM represents an important research gap.

Consistent with preclinical findings, CCM patients also have upregulated levels of the fecal bacterial LPS synthesis pathway and higher relative abundance of distinct bacterial species that are associated with increased disease severity [14] (Table 1). Shotgun metagenomics on fecal samples revealed substantial differences in both alpha and beta diversity in CCM patients compared to healthy individuals, indicating a significant alteration in bacterial communities associated with the disease [14]. The abundance of GNB, specifically *Odoribacter splanchnicus*, was markedly higher in the CCM patients, while gram-positive bacteria (GPB) such as *Faecalibacterium prausnitzii* and *Bifidobacterium adolescentis* were significantly reduced in the CCM patients compared to the healthy group [14]. This resulted in an altered ratio between GNB and GPB in the CCM patients. Moreover, the study observed an upregulation of genes related to LPS synthesis in the CCM patients, accompanied by a significant decrease in plasma levels of lipopolysaccharide-binding protein (LBP) [14]. Notably, several bacterial species altered in CCM patients and murine models are also implicated in other neurological diseases. Increased *Odoribacter splanchnicus* and decreased *Faecalibacterium prausnitzii* have been associated with inflammation and neurodegeneration in Alzheimer’s and Parkinson’s diseases [30], while shifts toward Gram-negative bacteria have been linked to endothelial dysfunction in cerebral small vessel disease. These shared microbial patterns suggest common gut–brain axis mechanisms across neurovascular and neuroinflammatory disorders.

**Figure 1:**
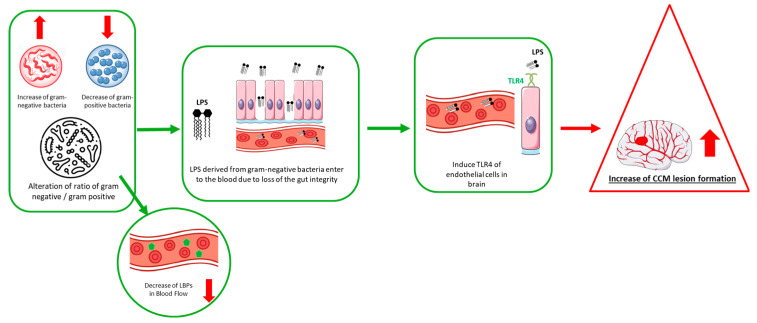
Schematic diagram demonstrating the existence of the gut–brain axis and CCM disease.

The gut microbiome changes in CCM patients were further explored by Srinath et al. in 2023 by examining plasma metabolome disparities between CCM patients and healthy individuals [15]. The study identified a significant correlation between gut microbiome alterations and changes in plasma metabolites among the CCM patients. Elevated plasma concentrations of cholic acid and hypoxanthine were observed, corresponding with increased expression of genes in *Bifidobacterium adolescentis*, *Faecalibacterium prausnitzii*, and *Odoribacter splanchnicus*. Cholic acid may exacerbate intestinal and systemic inflammation through the activation of bile acid receptors such as FXR, thereby potentially disrupting vascular permeability [32]. Meanwhile, hypoxanthine serves as an indicator of oxidative stress, which can compromise endothelial integrity [15]. Pathway analysis further demonstrated that cholic acid and hypoxanthine are involved in key CCM-related signaling pathways, such as PI3K-AKT, MAPK, NF-KB, and Rap [15]. These pathways are critical in regulating cellular processes like inflammation, cell survival, and immune response, suggesting that metabolomic shifts driven by gut microbiome alterations may play a central role in CCM pathogenesis and disease progression. Comparative studies reveal that both murine models and human CCM patients exhibit gut microbiota alterations characterized by an increase in Gram-negative, LPS-producing bacteria and a decrease in beneficial Gram-positive species. In murine models, activation of endothelial TLR4 by LPS from Gram-negative bacteria, such as those from the *Bacteroidetes* phylum, has been shown to promote CCM lesion formation [14,19,20,31]. Conversely, germ-free mice or those treated with antibiotics that reduce Gram-negative bacterial load exhibit a significant reduction in lesion development. In human CCM patients, increased abundance of Gram-negative bacteria like *Odoribacter splanchnicus* and decreased levels of anti-inflammatory Gram-positive bacteria such as *Faecalibacterium prausnitzii* and *Bifidobacterium adolescentis* have been reported. Importantly, these microbiome alterations have been reported in both familial (genetic) and sporadic CCM patients, with some taxa showing differences based on germline mutation status—for example, enrichment of *Clostridiales*, *Bacteroides*, and *Prevotella* has been observed in familial cases compared to sporadic ones [14,31]. Hence, these studies have highlighted an important link between the gut microbiome and systemic metabolic processes, further implicating microbial dysbiosis in the pathophysiology of CCM (Figure 1).

Collectively, these studies suggest that the altered gut microbiome could potentially alleviate or exacerbate CCM lesion development and progression as well as highly variable clinical outcomes (e.g., headaches, neurological deficits, seizures, stroke). These changes in the gut microbiome may serve as a third hit in CCM pathogenesis by modulating immune response and inflammatory process, which are implicated in CCM pathogenesis [19,23]. However, the role of the gut microbiome in CCM is an emerging area of research that requires further systematic investigations to fully understand its potential impact on the development and progression of CCM disease. Nevertheless, identification of the gut–brain axis in CCM unveiled a new novel therapeutic strategy for CCM by targeting the gut microbiome.

Targeting the gut microbiome using antibiotics and a germ-free environment in mouse models has shown promising results in preventing CCM lesion development [19,20]. However, there needs to be more targeted gut microbiome therapy to solve the unmet clinical need of CCM patients. Innovative approaches of targeting the gut microbiome (e.g., diets, prebiotics, probiotics) have been tested and readily used in clinics with well-known safety profiles in patients [33]. Yet, these putative options have not been considered in CCM patients. This is largely due to our limited understanding and efforts in testing the gut microbiome modification strategies in CCM disease.

## 3. Gut–Brain Axis

The communication network between the gastrointestinal tract (GIT) and central nervous system involves a complex interplay of intestinal epithelial cells (enterocytes and goblet cells), enteroendocrine cells (EECs), enteric neurons, smooth muscle cells, interstitial cells of Cajal (ICCs), enteric glial cells (EGCs), and resident immune cells such as macrophages and dendritic cells, together with neurotransmitters, hormones, and cytokines. This phenomenon is referred to as the “gut–brain axis” [34,35]. This bidirectional communication system plays a critical role in maintaining physiological homeostasis and influencing various neurological processes, and it affects brain function, behavior, and pathology, and vice versa [36,37,38,39].

Recent studies revealed that the gut microbiome actively participates in the gut–brain axis by producing neurotransmitters, such as serotonin, gamma-aminobutyric acid (GABA), and dopamine, as well as other bioactive molecules [37,40]. These microbial-derived compounds can influence brain function and behaviors through two primary pathways. Firstly, they act locally on the enteric nervous system (ENS), which directly communicates with the brain via the vagus nerve [41,42]. Secondly, they can exert systemic effects by entering the bloodstream and crossing the blood–brain barrier, thereby influencing brain activity and potentially impacting mood, cognition, and stress responses [43]. In CCM specifically, gut-derived lipopolysaccharide (LPS) has been shown to activate endothelial TLR4 signaling, directly contributing to lesion formation in murine models [19]. Moreover, human CCM patients exhibit distinct gut microbiota profiles correlated with disease severity, suggesting that gut–brain axis dysfunction may influence CCM progression [14]. Parallels with other neurovascular and neuroinflammatory diseases support the potential role of gut–brain interactions in CCM: in cerebral small vessel disease, gut dysbiosis contributes to endothelial dysfunction and cognitive impairment [44]; in multiple sclerosis, gut microbiota changes modulate CNS inflammation [45]; and in stroke, alteration in the gut microbiota worsens blood–brain barrier breakdown [46].

For example, gastrointestinal complications, such as constipation, are frequently observed in children having autistic spectrum disorders (ASD). A metagenomics study examining the gut microbiome profile of children with ASD revealed an increased *Firmicutes* to *Bacteroidetes* ratio, along with a higher abundance of facultative anaerobic bacteria such as *Escherichia, Shigella*, and *Candida* fungi. These microbial imbalances may contribute to the gastrointestinal issues commonly experienced by children with ASD [47,48]. These findings link childhood autism to alterations in gut microbiome composition and their metabolites, suggesting potential changes in the neuroimmune and neuroendocrine systems in affected individuals. This connection highlights the possibility that gut microbiota imbalances may play a role in the neurological and immune-related characteristics of ASD [49].

Another key piece of evidence supporting the influence of the gut microbiome on the CNS is its role in producing GABA. GABA is a primary inhibitory neurotransmitter in the brain, responsible for slowing down neuronal communication. This regulation can help alleviate symptoms associated with various neurological disorders, such as depression and anxiety. The ability of the gut microbiome to modulate GABA production highlights the potential for microbiota-based interventions in managing such conditions [50]. Metagenomic analyses have also revealed that *Faecalibacterium* and *Coprococcus*, known for their production of butyrate, are associated with positive effects on mental health in individuals suffering from depression [51]. In contrast, the genera *Dialister* and *Coprococcus* were found to be absent in gut microbiome samples from individuals with severe depression, compared to healthy controls. These findings suggest a potential link between butyrate-producing bacteria and mental health, highlighting the role of the gut microbiome in modulating mood and psychiatric conditions [51].

Dietary interventions have the potential to influence neurological conditions by altering the gut microbiome, further supporting the concept of the gut microbiome–brain axis. A well-established example of the interaction between diet, the microbiome, and human health is the development of atherosclerosis in both the cerebral and coronary vessels. The reduced arterial diameter caused by plaque formation can result in significant cardiovascular and cerebrovascular complications. Impaired blood flow increases the risk of severe events such as heart attacks, strokes, and peripheral artery disease [52].

Diets rich in animal proteins and fats contain significant levels of phosphatidylcholine (PC), a specific type of lipid that undergoes microbial hydrolysis in the gut. This process converts PC into trimethylamine (TMA), a gas that enters the bloodstream and is subsequently transported to the liver. In the liver, TMA is oxidized into trimethylamine N-oxide (TMAO), a metabolite increasingly recognized for its role in promoting vascular pathology [53]. Research has shown that mice fed with a high-PC-content diet exhibit elevated levels of TMAO in their blood serum, which correlates with an increased susceptibility to atherosclerosis [54,55]. Elevated TMAO levels contribute to the formation of vascular plaques by promoting cholesterol deposition in the arterial walls and impairing cholesterol clearance [56]. This highlights the significant role that dietary components and gut microbiota interactions play in cardiovascular health, suggesting that dietary modification may offer a therapeutic approach to reduce atherosclerotic risk through the modulation of TMAO production.

Another example of diet-induced regulation of the gut–brain axis is shown in ketogenic diet studies. A ketogenic diet is characterized by its high content of plant-based fats and proteins and minimal carbohydrates, and based on results from a randomized controlled trial, the ketogenic diet has been suggested as an effective therapeutic intervention for pediatric epilepsy [57]. This diet induces a metabolic state of ketosis, in which the body primarily relies on fats for energy rather than glucose. Studies have shown that the ketogenic diet can significantly reduce the frequency and severity of seizures in children having drug-resistant epilepsy, making it a valuable non-pharmacological treatment option. Its efficacy is thought to stem from changes in energy metabolism, along with possible effects on the gut microbiome and neuronal activity [58,59]. The effects of a ketogenic diet have also been explored in the context of various neurological diseases. Research has provided growing evidence that a ketogenic diet may have a beneficial impact on these conditions, potentially improving cognitive function in Alzheimer’s disease (AD) and motor symptoms in Parkinson’s disease. Despite these promising findings, the precise mechanisms through which the ketogenic diet exerts its positive effects remain unclear. Proposed mechanisms include enhanced mitochondrial function, reduced oxidative stress, anti-inflammatory effects, and modulation of the gut microbiome, but further studies are needed to fully elucidate the pathways involved in these neuroprotective actions [60,61].

In addition to dietary modifications, supplementation with probiotics and prebiotics can also influence the gut microbiome and potentially enhance CNS conditions. The term “psychobiotics” is used to describe probiotics and prebiotics that have shown promise in preclinical studies for their ability to impact neurological disorders and alleviate cognitive symptoms associated with conditions such as anxiety, depression, epilepsy, and AD. These supplements may exert their effects by modulating the gut microbiota, thereby influencing brain function and behaviors through regulating the gut–brain axis. However, further research is needed to fully understand the therapeutic potential and mechanisms of psychobiotics in treating these conditions [42,62,63,64]. These examples illustrate how gut–brain axis dysfunction can impact neurovascular integrity, reinforcing its potential relevance in CCM.

## 4. Gut Microbiome Manipulations

The gut microbiome composition can be manipulated through a range of factors (Table 1). These include lifestyle changes such as environment, dietary changes, physical activity levels, smoking, drug use (e.g., antibiotics), and stress [65,66]. Although each individual has a unique gut microbiome profile in terms of bacterial diversity and abundance—with notable differences by age group and sex (male and female)—people with similar lifestyles and living environments tend to share comparable gut microbiome enterotypes [67,68,69,70]. This suggests that environmental and lifestyle factors are pivotal in shaping the gut microbiome, despite individual variability. As previously highlighted, the baseline gut microbiome profile is a key determinant in the trajectory of microbial community changes. Beyond dietary interventions, there are several other approaches aimed at promoting a more favorable or “targeted” microbiome composition. These include fecal microbiota transplantation (FMT) and the strategic use of prebiotics and probiotics to support the growth of beneficial microorganisms. Hence, in this section, we have reviewed various strategies that can manipulate the gut microbiome (Table 2).

### 4.1. Diets

**Figure 2:**
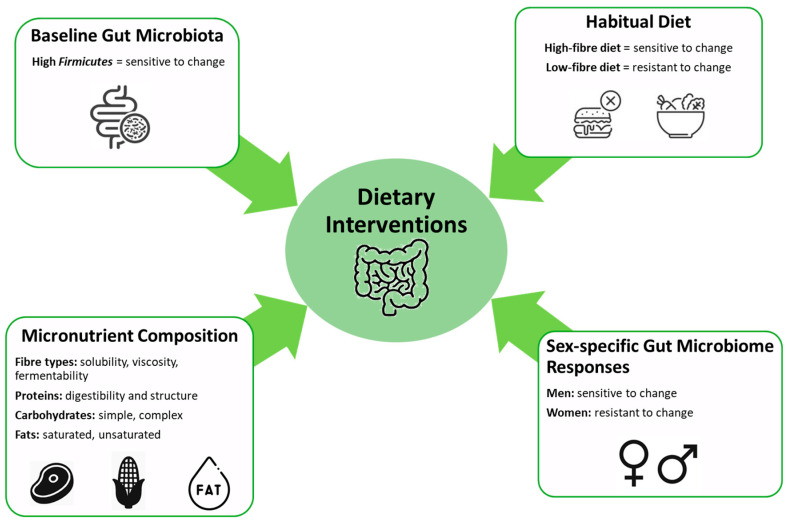
Key factors influencing gut microbiome responses to dietary interventions.

The gut microbiome refers to the collection of microorganisms, encompassing bacteria, viruses, fungi, and protozoa, which inhabit the GIT of humans and other animal species [71,72]. The gut microbiome plays a vital role in digestion, immunity, and overall health [73]. Diet significantly impacts the composition and function of the gut microbiome, and changes in the gut microbiome can affect how the body processes food and nutrients (Figure 2). Extensive evidence indicates that the communities of microorganisms residing in the gut can produce a variety of metabolites that have profound impacts on human health and disease states. These metabolites can influence systemic conditions, including the manifestations of inflammatory and autoimmune diseases [74,75].

Baseline gut microbiota composition, specifically when it includes a high abundance of Firmicutes, is sensitive to dietary changes [76]. Habitual diet type significantly impacts microbiome adaptability, with high-fiber diets being more responsive compared to low-fiber diets [77]. Micronutrient composition, including the types of fiber (solubility, viscosity, fermentability), proteins (digestibility, structure), carbohydrates (simple, complex), and fats (saturated, unsaturated), critically modulates microbiome alterations [78]. Additionally, sex-specific differences are noted, with men exhibiting greater microbiome responsiveness to dietary modifications compared to women [79]. A study of twins’ gut microbiome profile by metagenomics analysis revealed that environmental influences such as diet and living conditions could have a major impact on gut microbiome diversity and abundance [80]. The interaction between diet and the gut microbiome is a complex and bidirectional relationship, with significant implications for human health. For instance, undernutrition remains a critical health challenge, contributing to 45% of childhood mortality and higher morbidity rates, particularly in sub-Saharan Africa and South Asia [81]. Gordon and colleagues hypothesized a model illustrating the intricate relationships between the gut microbiota, the immune system, and diet, which collectively contribute to the development of malnutrition [82]. Subsequent studies have provided robust evidence supporting this model, demonstrating that gut microbiota dysbiosis is causally linked to childhood undernutrition [83,84]. Furthermore, research in Gambia highlighted a significant interaction between the gut microbiome composition of infants and the levels of human milk oligosaccharides (HMO) in maternal breast milk, which was associated with reduced infant morbidity [85]. These findings underscore the critical role of the gut microbiome in mediating the effects of diet on health outcomes, particularly in vulnerable populations. Although there are currently no studies directly investigating the impact of dietary interventions on CCM progression, evidence from cerebral small vessel disease suggests that Mediterranean-style diets can improve endothelial function and reduce neurovascular inflammation [86], which may have implications for CCM given shared microvascular pathophysiology.

Given the close relationship between gut microbiome composition and dietary intake, a pertinent question arises: can we modulate the gut microbiome through specific dietary interventions to alleviate the clinical symptoms of diseases or decrease the likelihood of predisposition to disease? The answer is not straightforward, as the gut microbiome is highly individualized, influenced by factors such as age, geographic location, and lifestyle. Nevertheless, dietary interventions hold significant potential to induce substantial shifts in the gut microbiome profile. Currently, there are no meta-analyses examining diet as a risk factor for CCM. Future epidemiological studies and systematic reviews are needed to determine whether dietary patterns influence CCM development or progression.

To explore this potential, it is essential first to investigate the impact of major dietary components on gut microbiome profile. This analysis should extend to understanding how these changes in microbiome profiles correlate with various disease conditions. By systematically assessing the influence of different dietary components, such as macronutrients (fibers, fats, and proteins) on the gut microbiome, we can begin to elucidate the complex interactions between diet, microbial ecology, and disease outcomes. Ultimately, such research could pave the way for personalized dietary strategies aimed at modulating the gut microbiome to prevent or mitigate disease.

Although microbial enterotypes are strongly influenced by long-term habitual diets, diet alone is not the sole determinant of gut microbiome composition. Baseline gut microbiota play a critical role in how individuals respond to dietary interventions. For instance, one study demonstrated that alterations in the gut microbiome composition in obese men following high-fiber diet interventions, such as resistant starch and non-starch polysaccharides, could be predicted based on the baseline microbial community structure, particularly the abundance of *Firmicutes* [87].

Furthermore, individuals with a habitual high-fiber diet showed greater sensitivity to changes induced by inulin-type fructan prebiotic interventions compared to those having low fiber consumption, whose gut microbiomes were more resistant to change [88]. While some studies have shown that alterations in the gut microbiome can occur within days or weeks of dietary intervention, the macronutrient composition of the diet, including both the quantity and quality of dietary components and their ratios, is crucial. For example, dietary fibers vary in structure, solubility, viscosity, and fermentability, and each type exerts distinct effects on the gut microbiome [89].

Additionally, each kind of protein also influences microbiome composition in different ways; for example, casein, a slowly digestible animal-derived protein, was found to be the most effective in preventing weight gain and fat accumulation in mice compared to a diet rich in red meat proteins, which are associated with obesity and vascular diseases [90]. Another important factor influencing gut microbiome composition is sex-specific microbial responses. Recent studies have shown that changes in the gut microbiome related to insulin sensitivity were observed in men but not in women [91]. Moreover, dietary interventions that combined antioxidants such as epigallocatechin gallate (EGCG) and resveratrol also resulted in alterations in the gut microbiome in men but not in women [92]. The development of targeted, precision diet interventions and optimized macronutrient ratios to manipulate the gut microbiome to alleviate clinical symptoms or prevent disease is a promising area of research. However, additional studies are needed to fully understand the complexities involved and to develop effective strategies.

Although there are currently no studies directly assessing dietary interventions in CCM, the established role of gut-derived lipopolysaccharide and endothelial inflammation in CCM pathogenesis suggests that specific dietary strategies could have therapeutic potential. Diets rich in fermentable fibers, polyphenols, and prebiotic compounds, such as Mediterranean-style diets, have been shown in other neurovascular diseases to promote anti-inflammatory gut bacteria (e.g., *Faecalibacterium prausnitzii*) and reduce circulating LPS levels, which could, in theory, mitigate endothelial dysfunction and CCM lesion progression. Future research should explore the effects of precision dietary interventions targeting gut dysbiosis in CCM patients or murine models, including macronutrient optimization and the use of dietary components having proven microbiota-modulating properties.

### 4.2. Faecal Microbiota Transplantation (FMT)

FMT is a medical procedure in which stool from a healthy donor is introduced into the GIT of a recipient to re-establish a balanced gut microbiota. This procedure has proven to be an effective treatment for recurrent *Clostridioides difficile* infections, a bacterial infection that leads to severe diarrhea and can pose life-threatening risks. FMT works by restoring healthy bacterial populations, which helps combat the overgrowth of harmful bacteria like *C. difficile* [93].

Previous studies investigating the gut microbiome in both healthy individuals and patients with various neurological disorders, such as Parkinson’s disease, multiple sclerosis, ASD, AD, Rett syndrome, neuromyelitis optica, amyotrophic lateral sclerosis, and epilepsy, have revealed significant differences in the diversity and abundance of bacterial communities in the gut [94,95,96,97,98,99,100,101,102]. Patients with these neurological disorders often exhibit gastrointestinal issues, which may indicate the involvement of the GIT in disease pathogenesis [103,104]. In both animal models of ASD and clinical studies involving ASD patients, gut microbiome interventions using probiotics have been shown to alleviate neurological and gastrointestinal symptoms. Specifically, probiotic treatments have been linked to reductions in anxiety, improved focus, and better management of gastrointestinal issues such as constipation and diarrhea [105,106,107].

In a study involving germ-free mice, FMT from donors with ASD resulted in the mice and their offspring displaying ASD-like symptoms, suggesting a direct link between gut microbiome composition and the manifestation of ASD traits. Additionally, another study using an ASD hamster model found a significant reduction in cerebral oxidative stress following FMT from healthy hamsters. This effect was further enhanced when *Lactobacillus paracasei* was administered as a probiotic, indicating the potential therapeutic role of both FMT and specific probiotics in mitigating ASD-related neurological symptoms [108,109].

In post-stroke patients, alterations in gut microbiome composition have been strongly linked to stroke severity. Stroke-induced reductions in GI motility promote the overgrowth of certain bacterial species and reduce microbial diversity. This gut dysbiosis compromises the integrity of the intestinal barrier, leading to the infiltration of pro-inflammatory immune cells into lymphoid tissues. Additionally, bacterial components or metabolites translocate into the bloodstream and ultimately reach the brain [46,110]. In an experimental study using a mouse model of stroke, mice treated with antibiotics following stroke exhibited a higher mortality rate compared to the control group. However, when these mice received FMT from specific pathogen-free donors, their mortality rate and infarct volume were like those of the control group. These findings suggest that altering the gut microbiome through antibiotic administration may increase post-stroke mortality [111].

Recent studies have demonstrated that the gut microbiome composition in AD patients is markedly different from that of healthy elderly individuals [98,112,113,114,115]. In AD patients, there has been a documented increase in LPS levels and a higher abundance of certain bacterial populations [116,117]. Increased levels of LPS contribute to inflammation through endotoxin mechanisms, which are associated with amyloid neurotoxicity and neuroinflammation. This inflammatory response is believed to play a critical role in the progression of AD [118,119]. Two notable studies have explored gut microbiome interventions via FMT in AD mouse models. In one study, broad-spectrum antibiotics were administered to deplete the gut microbiome in transgenic AD mice. This intervention led to a reduction in amyloid-beta 42 (Aβ42) accumulation and neuroinflammation, though these effects were observed exclusively in male mice.

In contrast, another study involving Aβ-treated mice receiving FMT from non-Aβ-treated mice only partially restored the levels of Aβ42 and neuroinflammation, suggesting that while FMT may have a mitigating effect, it does not fully reverse the pathological changes associated with AD [120]. Another study involving germ-free AD mice demonstrated that FMT from AD mice versus normal mice led to distinct outcomes in the progression of AD in the germ-free recipients. FMT from AD mice exacerbated AD symptoms in the germ-free mice, while FMT from healthy, non-AD mice did not significantly impact or even potentially mitigated the progression of AD. These results highlight the differential effects of gut microbial manipulation through FMT on AD pathology and suggest that the composition of the gut microbiome plays a crucial role in modulating the disease progression. Alterations in the gut microbiota, either through depletion or transplantation, can influence key aspects of AD, such as neuroinflammation and amyloid-beta accumulation. This underscores the importance of the gut–brain axis in AD and indicates that targeted manipulation of the gut microbiome may offer a promising therapeutic approach to influence the course of the disease. Although no studies have yet investigated fecal microbiota transplantation (FMT) in the context of CCM, evidence from neurological disease models such as autism spectrum disorder, stroke, and Alzheimer’s disease indicates that gut microbiome manipulation via FMT can significantly impact neuroinflammation, blood–brain barrier integrity, and the disease progression. Given the established role of gut-derived lipopolysaccharide and TLR4 activation in CCM lesion formation, future research could apply FMT in CCM murine models to directly assess whether transferring dysbiotic microbiota from CCM patients induces or exacerbates lesion development, providing critical insights into the causal role of the gut microbiome in CCM pathogenesis. While the potential therapeutic application of FMT in CCM presents an intriguing non-surgical intervention that could reduce symptoms by altering the gut microbiome, rigorous preclinical and clinical studies are essential to establish its safety, efficacy, and optimal protocols. Challenges include patient selection, donor screening, standardization of transplantation procedures, variability in individual responses, long-term impacts on the gut microbiota, and the risk of infectious agent transmission. Furthermore, ethical considerations, regulatory hurdles, and logistical complexities must be carefully addressed before FMT can be considered a viable therapeutic strategy for CCM.

### 4.3. Prebiotics and Probiotics

Prebiotics are non-digestible fibers that selectively promote the growth of beneficial gut bacteria. These compounds, including inulin, fructooligosaccharides, galactooligosaccharides, resistant starch, beta-glucan, and pectin, serve as substrates for commensal bacteria, enhancing their activity. Prebiotics facilitate the growth of bacteria that produce SCFAs, such as butyrate, which has been associated with improved gut health and a reduced risk of inflammatory diseases [121]. Probiotics, on the other hand, are live microorganisms that provide health benefits when consumed in sufficient quantities. Common probiotic strains include those from the *Lactobacillus* and *Bifidobacterium* genera, both recognized for their positive effects on gut health [122,123]. Research has shown that probiotics may be effective in managing GI disorders such as irritable bowel syndrome, inflammatory bowel disease, and antibiotic-associated diarrhea. Additionally, probiotics have been studied for their immune-modulating properties and their potential to reduce inflammation in both the gut and other areas of the body.

Prebiotics and probiotics work synergistically, with prebiotics serving as the nutritional foundation for probiotics [124]. A combination of probiotics and prebiotics (synbiotics) creates a balanced gut ecosystem, contributing to enhanced overall health and well-being. While studies administering probiotics in CCM models or patients are lacking, animal studies in multiple sclerosis have shown that certain probiotic strains can reduce neuroinflammation [125], suggesting that similar approaches could be explored in CCM to modulate endothelial dysfunction.

Several studies have indicated that post-stroke synbiotic supplement regimens can reduce inflammation and neurological deficits, as well as improve memory and learning in murine models of stroke [126,127]. Additionally, evidence suggests that synbiotics can lower the risk of cardiovascular disease by reducing blood cholesterol levels and influencing the production of TMAO, a compound implicated in atherosclerosis formation [128]. Moreover, treatment with various combinations of probiotics and synbiotics before, during, and after chemotherapy in cancer patients has shown varying degrees of alleviation in therapy-related complications [129]. Although these findings highlight the potential benefits of synbiotic interventions, they are not yet considered targeted or disease-specific therapeutic strategies for improving health conditions. By selecting specific prebiotics or carefully balancing prebiotic ratios, it is possible to promote the growth of beneficial bacterial genera that may have diminished or disappeared due to disease conditions or invasive therapies, thereby enhancing microbiome diversity.

Certain prebiotics also have the capacity to suppress or reduce the abundance of pathogenic bacteria that tend to flourish during disease onset by fostering the growth of other commensal bacteria [130]. When these prebiotics are combined with targeted probiotics—bacterial genera that have been identified through deep metagenomic analysis as being depleted or significantly reduced in patients with a particular non-invasive disease—targeted synbiotic therapy could be developed. This approach has the potential to restore microbial balance and improve patient outcomes by addressing specific microbial deficiencies associated with disease. Extensive additional research is required to design tailored interventions that effectively target specific diseases. This includes a deeper understanding of the complex interactions between the gut microbiome and various disease states, as well as identifying the most appropriate prebiotic and probiotic combinations that can restore microbial balance and enhance health outcomes in a disease-specific manner.

Targeted gut microbiome modification using synbiotics represents a potentially therapeutic intervention in CCM and associated vascular disorders. While probiotics classically fortify gut integrity, new data indicate systemic activities involving regulation of inflammation and oxidative stress and intestinal barrier integrity, each involved in CCM pathophysiology. Prebiotics promote desired bacteria growth and, in synbiotics and selected probiotics, enhance balance to the gut ecosystem and potentially influence CCM lesion formation. Through the use of metagenomic data to identify strains having established preclinical efficacy or common to healthy microbiomes, individualized synbiotics can tighten the intestinal barrier, reduce translocation of pro-inflammatory agents such as LPS, and provide desirable regulation to lipid and glucose metabolism. These several activities may repress systemic inflammation and endothelial malfunction and otherwise regulate CCM lesion formation and growth. Currently, no studies have assessed the administration of probiotics and prebiotics in CCM patients or animal models. Given the role of gut dysbiosis and LPS-mediated endothelial activation in CCM pathogenesis, future research should investigate whether specific probiotic strains can reduce Gram-negative bacterial abundance, decrease systemic inflammation, and attenuate lesion formation.

## 5. Summary and Future Directions

Despite the significant progress in elucidating the mechanisms that are responsible for CCM disease, CCM patients still lack targeted therapies and are limited to high-risk neurosurgery. This is primarily due to the identified therapeutic targets (e.g., TLR4, MEKK3, KLF2/4) having far broader roles in vascular homeostasis, so targeting these molecules can lead to severe side effects especially in children, outweighing any benefits. Hence, they are not ideal therapeutic targets for CCM disease, and new therapeutic strategies are urgently needed. Genetic mutations, particularly in the CCM1, CCM2, and CCM3 genes, have been recognized as key elements in the initiation of lesion formation. However, the role of non-genetic factors has emerged as a potential contributor to the disease.

Emerging evidence suggests that additional non-genetic factors, such as environmental influences and lifestyle choices, may significantly impact both the formation and severity of CCM lesions. This indicates a multifactorial etiology in which genetic predisposition interacts with external triggers, such as oxidative stress, inflammatory processes, or changes in the gut microbiome. These findings point to a complex interplay between genetics and environmental factors, emphasizing that CCM pathology likely results from a dynamic and multifaceted process, rather than a singular genetic cause. Understanding this intricate relationship will be crucial for developing more effective therapeutic interventions and diagnostic strategies for CCM patients.

The use of targeted synbiotics for CCM is still in its early research stages, but their potential to influence vascular homeostasis offers promising prospects such as a non-invasive adjunct therapy. However, comprehensive preclinical and clinical studies are essential to confirm the safety, efficacy, and underlying mechanisms by which specific combinations of probiotics and the appropriate ratio of prebiotics could benefit CCM patients. Further investigation is needed to determine the most suitable bacterial strains, types of prebiotics, optimal dosages, and treatment durations required for achieving the best therapeutic outcomes.

Despite emerging associations, evidence remains preliminary, and causality is yet to be established. The absence of direct dietary, FMT, probiotic, or synbiotic intervention studies in CCM is a critical gap. To advance the field, several key areas merit focused investigation:

1. **Characterization of baseline microbiome profiles in CCM disease:** A fundamental step involves identifying and characterizing the baseline gut microbiome composition in CCM patients compared to healthy individuals. Such comparative analyses may reveal disease-specific microbial signatures or dysbiotic patterns that could serve as diagnostic or prognostic indicators and guide targeted interventions.
2. **Microbiome-targeted therapeutics—probiotics, synbiotics, and diets:** Integrative strategies employing specific probiotic strains, rationally designed synbiotic combinations, and precision nutrition approaches should be tested for their capacity to attenuate gut-derived inflammation and modulate host immune responses. These strategies should particularly focus on reducing Gram-negative bacterial taxa and associated endotoxins, which are hypothesized to contribute to endothelial dysfunction in CCM pathogenesis.
3. **Bridging preclinical and clinical research—translational research**: Longitudinal studies involving both well characterized animal models and patient cohorts are needed to evaluate causal links between microbiome shifts and CCM outcomes. Integration of multi-omics approaches—such as metagenomics, metabolomics, and transcriptomics—will enhance mechanistic understanding and enable the identification of therapeutic windows.

Moving forward, robust preclinical validations are required using well-characterized CCM animal models that closely reflect the human disease. Effective translation will depend not only on careful experimental design and standardized microbiome analysis techniques but also on coordinated, multidisciplinary collaboration across microbiology, neurology, immunology, nutrition science, and clinical trial methodology. In parallel, regulatory considerations and consensus on microbial strain identification, dosage, and intervention protocols will be necessary to support reproducibility. Systematically addressing these challenges is essential to transform early laboratory discoveries into reliable, safe, and effective therapeutic options for individuals living with CCM.

## Figures and Tables

**Table 1:** Comparative analysis of gut microbiota alterations in murine CCM models and human CCM patients.

Model/Population	Key Alterations in Gut Microbiota	Implications	Reference
Murine Models	**↑** Gram-negative *Muribaculaceae* (*Bacteroidetes* s24-7)	Activates endothelial TLR4 signaling, promoting CCM lesion formation. Antibiotic-treated or germ-free mice show reduced lesion burden.	[19]
Human CCM Patients	↑ Gram-negative *Odoribacter splanchnicus* ↓ Gram-positive *Faecalibacterium prausnitzii* ↓ Gram-positive *Bifidobacterium adolescentis*	Correlates with disease severity and genetic mutations; suggests gut microbiome may influence CCM progression and provide diagnostic biomarkers.	[14,31]

**Table 2:** Key influencers of gut microbiome composition.

Gut Microbiome Influencer	Factors
**Lifestyle**	- Environment - Dietary changes - Physical activity - Smoking - Drug use (e.g., antibiotics) - Stress - Shared lifestyles and environments resulting in similar gut enterotypes
**Biological Factors**	- Age - Individual uniqueness in bacterial diversity and abundance - Sex differences (men vs. women)
**Microbiome Profile**	- Determines response to interventions and microbial shifts
**Manipulation Strategies**	- Fecal microbiota transplantation (FMT) - Prebiotics/Probiotics - Dietary interventions (fibers, macronutrient ratios) - Disease-specific microbiome modulation - Precision nutrition approaches

## Abbreviations

AD, Alzheimer’s disease; ASD, Autistic spectrum disorders; Aβ42, amyloid-beta 42; CCM: cerebral cavernous malformation; CNS, central nervous system; DSS, dextran sulfate sodium; ECs, endothelial cells; EGCG, epigallocatechin gallate; ENS, enteric nervous system; FMT, fecal microbiota transplantation; GABA, gamma-aminobutyric acid; GIT, gastrointestinal tract; GNB, Gram-negative bacteria; GPB, Gram-positive bacteria; HMO, human milk oligosaccharides; KLF2/4, Krüppel-like factor 2/4; KRIT1, krev interaction trapped protein 1; LBP, lipopolysaccharide-binding protein; LPS, lipopolysaccharide; MEKK3, mitogen-activated protein kinase kinase kinase 3; MRI, magnetic resonance imaging; NGS, next-generation sequencing; PC, phosphatidylcholine; PDCD10, programmed cell death 10; SCFAs, short-chain fatty acids; TLR4, toll-like receptor 4; TMA, trimethylamine; TMAO, trimethylamine N-oxide.
